# Clinical failure with and without empiric atypical bacteria coverage in hospitalized adults with community-acquired pneumonia: a systematic review and meta-analysis

**DOI:** 10.1186/s12879-017-2495-5

**Published:** 2017-06-02

**Authors:** Khalid Eljaaly, Samah Alshehri, Ahmed Aljabri, Ivo Abraham, Mayar Al Mohajer, Andre C. Kalil, David E. Nix

**Affiliations:** 1 Department of Clinical Pharmacy, King Abdulaziz University, P.O. Box 80200, Jeddah, Postal code 21589 Saudi Arabia; 2 College of Pharmacy, University of Arizona, Drachman Hall – B306, 1295 N Martin Ave, P.O.Box 210202, Tucson, AZ USA; 3 Division of Infectious Diseases, Department of Medicine, University of Arizona, Tucson, AZ USA; 4 Department of Internal Medicine, Division of Infectious Diseases, University of Nebraska Medical Center, Omaha, NE USA

**Keywords:** Community-acquired pneumonia, Antibiotics, Atypical, Macrolides, Fluoroquinolones

## Abstract

**Background:**

Both typical and atypical bacteria can cause community-acquired pneumonia (CAP); however, the need for empiric atypical coverage remains controversial. Our objective was to evaluate the impact of antibiotic regimens with atypical coverage (a fluoroquinolone or combination of a macrolide/doxycycline with a β-lactam) to a regimen without atypical antibiotic coverage (β-lactam monotherapy) on rates of clinical failure (primary endpoint), mortality, bacteriologic failure, and adverse events, (secondary endpoints).

**Methods:**

We searched the PubMed, EMBASE and Cochrane Library databases for relevant RCTs of hospitalized CAP adults. We estimated risk ratios (RRs) with 95% confidence intervals (CIs) using a fixed-effect model, but used a random-effects model if significant heterogeneity (*I* ^*2*^) was observed.

**Results:**

Five RCTs with a total of 2011 patients were retained. A statistically significant lower clinical failure rate was observed with empiric atypical coverage (RR, 0.851 [95% CI, 0.732–0.99; *P* = 0.037]; *I* ^*2*^ = 0%). The secondary outcomes did not differ between the two study groups: mortality (RR = 0.549 [95% CI, 0.259–1.165, *P* = 0.118], *I* ^*2*^ = 61.434%) bacteriologic failure (RR = 0.816 [95% CI, 0.523–1.272, *P* = 0.369], *I* ^*2*^ = 0%), diarrhea (RR = 0.746 [95% CI, 0.311–1.790, *P* = 0.512], *I* ^*2*^ = 65.048%), and adverse events requiring antibiotic discontinuation (RR = 0.83 [95% CI, 0.542–1.270, *P* = 0.39], *I* ^*2*^ = 0%).

**Conclusions:**

Empiric atypical coverage was associated with a significant reduction in clinical failure in hospitalized adults with CAP. Reduction in mortality, bacterial failure, diarrhea, and discontinuation due to adverse effects were not significantly different between groups, but all estimates favored atypical coverage. Our findings provide support for the current guidelines recommendations to include empiric atypical coverage.

**Electronic supplementary material:**

The online version of this article (doi:10.1186/s12879-017-2495-5) contains supplementary material, which is available to authorized users.

## Background

Community-acquired pneumonia (CAP) is one of the leading causes of mortality [1–4]. This disease can be caused by a variety of pathogens, including typical and atypical bacteria. The most common typical bacteria causing CAP are *Streptococcus pneumoniae* and *Haemophilus influenzae*. The need to include empiric coverage for atypical bacteria, such as *Mycoplasma pneumoniae*, *Chlamydophila pneumoniae* and *Legionella spp.*, for all hospitalized adult patients is controversial. Adding antibiotics to cover atypical bacteria might increase the likelihood of adverse effects, bacterial resistance, and cost; However, routine empiric atypical coverage is recommended by the current major guidelines for CAP [1–4].

Prior meta-analyses of randomized clinical trials (RCTs) of atypical coverage for CAP have not demonstrated the benefit of empiric atypical coverage in the treatment of hospitalized adults with CAP. It should be noted, however, that these meta-analyses had major limitations despite including a large number of trials [5–7]. The two meta-analyses that found mortality benefit of empiric atypical coverage were based mainly on observational studies [8, 9].

The primary objective of this meta-analysis was to evaluate the impact of atypical coverage on rates of clinical failure with guideline-recommended antibiotic regimens. Rates of mortality, bacteriologic failure, and adverse events were evaluated as secondary outcomes. The meta-analysis was limited to RCTs comparing treatments with atypical coverage (a fluoroquinolone or combination of a macrolide/doxycycline with a β-lactam) to a regimen without atypical antibiotic coverage (β-lactam monotherapy).

## Methods

The meta-analysis was conducted according to the Preferred Reporting Items for Systematic Reviews and Meta-Analyses (PRISMA) guidelines (Additional file 1: Table S1).

### Search strategy and data extraction

An independent librarian helped to formulate the appropriate search strategy (provided in the Additional file 1). Two authors (S.A. and A.A.) independently searched the PubMed, Embase, and Cochrane Library biomedical databases without date restrictions through December 11, 2016 using a standard form for data extraction. Languages were limited to English, Spanish, Arabic, French, German, Italian, and Dutch. The references of included studies were checked to identify additional clinical trials. In addition, the ClinicalTrials.gov website was searched for unpublished trials through December 11, 2016. We only considered the results from the intention-to-treat analysis reported in each study. Any disagreement between the authors was resolved through discussion.

### Study selection

RCTs of hospitalized adult patients with CAP that compared empiric antibiotic regimens with atypical coverage (a respiratory fluoroquinolone or combination of a macrolide/doxycycline with a β-lactam) to a regimen without atypical antibiotic coverage (β-lactam monotherapy) were identified and included. The respiratory fluoroquinolones included levofloxacin, moxifloxacin, and gemifloxacin. The macrolides included azithromycin, clarithromycin, or erythromycin. β-lactam agents with >85% coverage against *S. pneumoniae* were allowed and this included amoxicillin, amoxicillin/clavulanate, ampicillin, ampicillin/sulbactam, piperacillin, piperacillin/tazobactam, cefuroxime, cefpodoxime, cefdinir, cefditorin, cefotaxime, ceftriaxone, cefepime, ceftaroline, imipenem, meropenem, and ertapenem. We excluded studies published as abstracts only; studies that deviated from the assigned empiric β-lactam monotherapy (permitted adding empiric atypical bacterial coverage); studies including >25% outpatients and/or >10% of patients with nosocomial pneumonia; and if the target population had conditions other than CAP but did not report separate outcomes for the CAP group.

### Data synthesis and analysis

The primary outcome was the rate of clinical failure of CAP. Secondary outcomes included rates of mortality, bacteriologic failure, and adverse events. Outcome rates assessed early during treatment or end of treatment were preferred over assessments at follow up post therapy. Heterogeneity (*I* ^*2*^) was assessed by using a Cochran’s chi-squared test. The risk ratios (RRs) with 95% confidence intervals (CIs) were estimated using fixed-effect models, but the random-effects models were used when significant heterogeneity between the studies was observed (*P*-value less than 0.1 in the chi-squared test for heterogeneity). We assessed the quality of studies by using the Cochrane risk of bias tool for RCTs (low, unclear or high) [10]. Funnel plot was used to evaluate publication bias, and this plot was provided as Additional file 1. All analyses were conducted using Comprehensive Meta-Analysis Version 3 software (Biostat, Englewood, NJ, USA).

## Results

### Search results

**Fig. 1:**
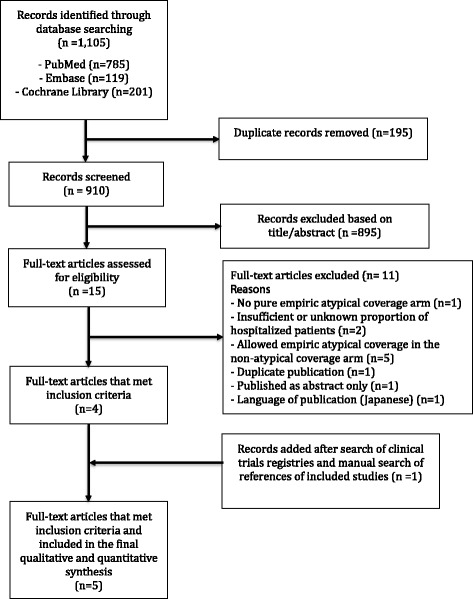
Flowchart of the process of literature search and extraction of studies meeting the inclusion criteria

The search process identified 1105 articles (PubMed 785; Embase 119; Cochrane Library 201) of duplicates for a total of 910 articles after removal [Fig. 1]. Fifteen full-text articles were assessed for eligibility after screening by title and/or abstract. After searching ClinicalTrials.gov and a manual search of references of included studies, five RCTs (with 2011 patients) were retained. A total of 998 patients treated with empiric atypical bacterial coverage were compared to 1013 patients treated without empiric atypical bacterial coverage.

### Study characteristics

The characteristics of the five included studies are summarized in Table 1. They were conducted between 1998 and 2014. Two studies were double-blinded [11, 12]; four were multicenter [11–14]; three were multinational and multicontinental [11–13]; one was funded by industry [13]; and four were in English [11–14] and one was in Spanish [15]. One study was excluded because of language (Japanese) [16]. The mean subject age in three studies was under 65 years [11, 12, 15]. Only one study included outpatients, who represented <25% of patients [12]. Two studies excluded patients with severe CAP [11, 15]. The regimen of β-lactams combined with a macrolide was used only in one study [14]. This study is the most recent one and included 580 patients). The other four studies included fluoroquinolones as the atypical bacterial coverage arm [11–13, 15]. The assessments of bias risk are summarized in the Additional file 1. All five studies were RCTs; the risks of selection bias (random sequence generation and allocation concealment) and detection bias (blinding of outcome assessment) were low in three studies; the risk of performance (blinding of participants and personnel) bias was low in two studies; and the risk of reporting bias was low in all studies.

**Table 1:** Characteristics of Included Studies

Study	Study Period; Publication Year	Design	Location	Funding Source	Enrolled Patients^a^; ITT	Age (years)	PNA Characteristics	Antibiotic Regimen^a^	Duration of Therapy (days)	Outcomes Definitions
Grain et al.	2009–2013; 2014	Non-inferiority open-label, RCT	6 sites in 1 country (Switzerland)	Non-industry	602; 580 (289 vs. 291)	76 (median)	Moderately severe PNA	BL (IV cefuroxime 1.5 G X 3/d or IV amoxicillin/ clavulanate 1.2 G X 4/d) vs BL + ML (IV/PO clarithromycin 500 mg X 2/d)	10	Mortality = 30-day. Failure = no clinical stability at day 7
Petitpretz et al.	1997–1998; 2001	Superiority, double-blind, RCT	82 sites in 20 countries (Europe, South America, Australia, Africa)	Non-industry	411; 408 (200 vs. 208)	51 (mean)	Mild-moderate PNA; suspected pneumococcal PNA; 79% hospitalized/21% outpatients	BL (PO amoxicillin 1 G X 3/d) vs FQ (PO moxifloxacin 400 mg X 1/d)	10	Mortality = during the study (38-day). Failure = no clinical/bacteriological response 3–5 days after end of therapy
Norrby et al.	Not reported; 1998	Superiority, open-label, RCT	64 sites in 13 countries (Europe, North and South America, Africa, Asia)	Industry	625; 619 (314 vs. 305)	65 (median)	Moderately severe PNA; excluded strongly suspected mycoplasma, chlamydia or legionella PNA; 94% CAP and 6% nosocomial PNA	BL (IV ceftriaxone 4 G X 1/d) vs FQ (IV levofloxacin 500 mg X 2/d, followed by PO levofloxacin 500 mg X 2/d)	8	Mortality = during the study (29-day). Failure = no clinical/bacteriological response 2–5 days after end of therapy
Leophonte et al.	1998–1999; 2004	Superiority, double-blind, RCT	102 sites in 3 countries (Europe, Africa)	Non-industry	324; 320 (167 vs. 153)	54 (mean)	Mild-moderate PNA; suspected pneumococcal PNA; 94% hospitalized	BL (PO amoxicillin/ clavulanate 1.2 G X 3/d) vs FQ (PO gemifloxacin 320 mg X 1/d)	7 for FQ; 10 for B-lactam	Mortality = during the study (30-day). Failure = no clinical/ bacteriological response at end of therapy
Kalbermatter et al.	1998; 2000	Superiority, open-label, RCT	1 site in 1 country (Argentina)	Non-industry	84; 84 (28 vs. 56)	60 (mean)	Mild-moderate PNA	BL (IV ceftriaxone 1 G X 2/d or IV amoxicillin/ clavulanate 1.2 G X 3/d) vs FQ (PO levofloxacin 500 mg X 2/d)	7–10 if favorable response	Failure = no clinical response at 72 h

^a^atypical bacterial coverage arm vs non-atypical bacterial coverage arm. *Abbreviations*: *PNA* pneumonia, *RCT* randomized clinical trial, *BL* β-lactam, *ML* Macrolide, *PO* orally, *IV* intravenously, *CAP* community-acquired pneumonia

### Primary outcome: clinical failure

**Fig. 2:**
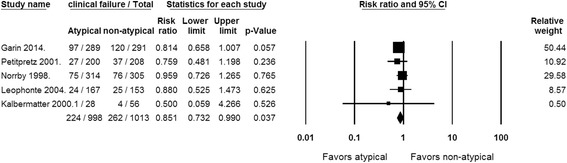
Forest plot showing the risk ratios of clinical failure for patients receiving empiric antibiotic therapy with versus without atypical coverage. Vertical line, “no difference” point between the 2 groups; horizontal line, 95% confidence interval; squares, risk ratios; diamonds, pooled risk ratios. Abbreviations: CI, Confidence interval

Clinical failure rates were reported in all 5 RCTs [Fig. 2]. Two of the studies reported early clinical outcomes at 72 h and 7 days. The remaining three trials assessed outcomes within a few days after end of treatment. A statistically significant lower clinical failure rate was observed with empiric atypical coverage (RR, 0.851 [95% CI, 0.732–0.99; *P* = 0.037]; *I* ^*2*^ = 0%; Q = 1.564 [*P* = 0.815]) in fixed-effect and random-effects model.

### Secondary outcomes: mortality, bacteriologic failure, and adverse events

**Fig. 3:**
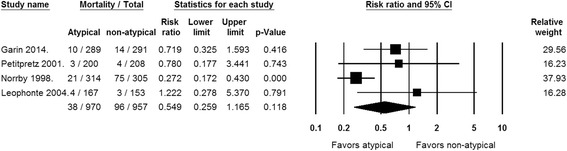
Forest plot showing the risk ratios of mortality for patients receiving empiric antibiotic therapy with versus without atypical coverage. Vertical line, “no difference” point between the 2 groups; horizontal line, 95% confidence interval; squares, risk ratios; diamonds, pooled risk ratios. Abbreviations: CI, Confidence interval

No statistical significance was identified with regard to any secondary outcomes. Mortality rates were reported in all studies [11–14] but one [14] [Fig. 3]. The rates of mortality, total adverse events, and diarrhea were analyzed using a random-effects model, while bacteriologic failure and adverse events requiring antibiotic discontinuation were analyzed using fixed-effect model. No statistically significant differences between the two regimens were observed in rates of mortality (RR = 0.549 [95% CI, 0.259–1.165, *P* = 0.118], *I* ^*2*^ = 61.434%; Q = 9.635 [*P* = 0.022]) bacteriologic failure (RR = 0.816 [95% CI, 0.523–1.272, *P* = 0.369], *I* ^*2*^ = 0%; Q = 0.47 [*P* = 0.79]), total adverse events (RR = 0.982 [95% CI, 0.697–1.383, *P* = 0.918], *I* ^*2*^ = 69.011%; Q = 5.722 [*P* = 0.057]), diarrhea (RR = 0.746 [95% CI, 0.311–1.790, *P* = 0.512], *I* ^*2*^ = 65.048%; Q = 6.454 [*P* = 0.04]), and adverse events requiring antibiotic discontinuation (RR = 0.83 [95% CI, 0.542–1.270, *P* = 0.39], *I* ^*2*^ = 0%; Q = 0.037 [*P* = 0.83]).

## Discussion

Our meta-analysis of RCTs confirms the benefit of empiric atypical bacteria coverage in hospitalized adult patients with CAP, unlike the other meta-analyses. This meta-analysis provides support for the current major guideline recommendations, including U.S guidelines of Infectious Diseases Society of America as well as European guidelines [1–4], using studies that used regimens recommended by these guidelines. The principal finding of our meta-analysis is that including empiric atypical coverage reduced the rates of clinical failure by approximately 15%. It should be noted that no single trial in our meta-analysis reported a statistically significant difference in the efficacy outcome, though there was a favorable trend in all 5 trials. However, the non-inferiority study by Garin et al., in which the empiric non-atypical bacterial coverage arm failed to meet the pre-specified non-inferiority threshold [14]. It is worth mentioning that a significant difference in clinical cure was found in previous meta-analyses favoring empiric atypical coverage in patients who had *Legionella* pneumonia [5, 6].

Our meta-analysis did not find a significant difference in mortality rates, which is consistent with other meta-analyses of RCTs [5–7]. Regimens that provided atypical coverage did not result in significantly more adverse events; however, adverse events were assessed in the studies involving respiratory fluoroquinolones and not in the macrolide-β-lactam combination study. The individual studies were not powered to detect differences in mortality and were not focused on adverse events. It remains unclear if adding empiric atypical coverage with a macrolide or doxycycline to a β-lactam increases the rate of adverse events. Future RCTs should evaluate benefits in terms of efficacy and potential harm in terms of adverse events and increased cost.

Our meta-analysis differs from prior meta-analyses of RCTs [5–7]. These meta-analyses included some studies of non-recommended comparators. For example, the inclusion of ciprofloxacin as monotherapy would be inappropriate due to poor activity against *S. pneumoniae*. The use of macrolide monotherapy may be inappropriate for the same reason and depending on the selected macrolide, coverage of *H. influenzae* may be poor. Studies of agents that have been withdrawn from the market, such as temafloxacin, have been included in these meta-analysis. Another limitation of prior meta-analyses is a focus on longer term outcomes (e.g. at 30 day follow up) and, therefore, any observed benefit could be attributed to confounding factors. The inclusion of studies that permitted adding empiric atypical coverage to the arm the should have lacked atypical coverage could bias the results against the benefit of including atypical coverage because it makes the two groups more similar and reduce our ability to assess the true benefit of empiric atypical coverage.

The stringent inclusion criteria make our meta-analysis unique, increases its clinical relevance, and addresses antibiotic regimens recommended in major CAP guidelines. Published studies including non-recommended and withdrawn antibiotics for hospitalized CAP adults were excluded to provide results that are relevant to clinical practice. In addition, we preferred clinical failure rates that were reported earlier rather than at the final assessment at post therapy follow up. Using outcomes collected at around day 30 post treatment allows for accumulation of confounding events including changes in therapy and evolution of underlying illness. For example, clinical failure rates in the Petitpretz et al. study [12] were 46/200 (23%) vs. 44/208 (21.2%) in two meta-analyses [5, 6] because they reported the rates during follow-up; the rates were 27/200 (12.2%) vs 37/208 (17.8%) in one meta-analysis [8] as well as ours when using rates reported at the end of therapy (rates difference, 1.8% vs 5.6%, respectively). RCTs should embrace early clinical outcome as an endpoint since this provides the most direct information about antimicrobial efficacy and improves discrimination of differences between treatments. The Food and Drug Administration’s 2014 guidance for developing drugs for treatment of community-acquired bacterial pneumonia stated that the time points at 36–48 h and 48–72 h after starting therapy demonstrate the greatest treatment effect of clinical recovery [17]. The guidance calls for a primary endpoint assessment on day 3 to day 5 of treatment.

Only five RCTs were found that meet our inclusion criteria. Despite the relatively small number of studies, subgroup analyses were performed for completeness and are available in the Additional file 1. Exclusion by language of publication can introduce bias and affects the results. However, only one study was excluded because of language in our meta-analysis [16]. Given the fact that the results of this study were available in an English abstract, we verified that including this study would not have altered the conclusions of our meta-analysis. Unfortunately, most RCTs have not reported detailed information about resistance rates, which is important to consider in studies of infectious diseases. Amoxicillin was used for typical coverage in one of the studies that we included and the coverage that this agent provides coverage that is inferior to that of moxifloxacin against *S. pneumoniae*, *H. influenzae* and *M. catarrhalis*. However, only one study included amoxicillin, in which all patients had their *H. influenzae* eradicated except three patients [13]. Amoxicillin is one of the recommended antibiotics per major guidelines and it is preferred over other excluded agents such as ciprofloxacin. Since moxifloxacin provides atypical coverage and better typical coverage, the treatment effect is not limited to the additional atypical coverage. Two of the studies included amoxicillin/clavulanate for typical bacterial coverage. The only deficiency here would be in coverage of penicillin non-susceptible *S. pneumoniae*; however, the incidence of these isolates in CAP studies is typically low [18–20]. In fact, this coverage deficit could be a problem when comparing any beta-lactam other than ceftaroline to a respiratory fluoroquinolone. If the goal is to evaluate impact of atypical coverage, then confounding factors need to be minimized. Therefore, a trial of ceftaroline versus ceftaroline plus a macrolide or respiratory fluoroquinolone would be valuable to sort out the effect of atypical coverage.

Etiologic diagnosis has evolved so that the pathogen can be identified in almost 90% of CAP cases [21]. In one study, atypical pathogens were detected in just over 4% of CAP cases [21]; however, there are outbreaks of *Legionella* spp. and areas with higher endemic incidence [22]. Given this low incidence, it is unlikely that any single RCT will ever be able to demonstrate the effect of atypical coverage for CAP. A better approach would be to include empiric atypical coverage for hospitalized (sicker) patients with CAP and then streamline therapy if the etiology is identified.

## Conclusions

Our restricted but targeted meta-analysis of RCTs was able to define a significant reduction (approximately 15%) in clinical failure with the inclusion of atypical coverage in hospitalized adults with CAP. No significant differences were found in terms of secondary outcomes including mortality, bacteriologic failure and adverse events. Our meta-analysis provides supports for the current recommendations of the major CAP guidelines. However, some of the difference noted may be due to differences in typical coverage between treatment arms.

## Additional file

Additional file 1: Search Strategy. **Figure S1.** Forest Plots for Bacteriologic Failure and Adverse Events in All Included Studies. **Figure S2.** Forest Plots for Subgroup Analyses of Clinical Failure. **Figure S3.** Funnel Plot of Included Studies for Clinical Failure. **Table S1.** PRISMA Check List. **Table S2.** Quality Assessment of Included Studies. **Table S3.** Characteristics of Excluded Full-Text Articles. (DOC 428 kb)

## Abbreviations

CAP: Community-acquired pneumonia
CI: Confidence interval
RCTs: Randomized clinical trials
RR: Risk ratio
