# Supplementary material for: Clinical failure with and without empiric atypical bacteria coverage in hospitalized adults with community-acquired pneumonia: a systematic review and meta-analysis
Source: BMC Infect Dis. 2017 Jun 2;17:385. doi: 10.1186/s12879-017-2495-5 (PMC5457549; doi:10.1186/s12879-017-2495-5)
Supplement: Additional file 1: — Search Strategy. Figure S1. Forest Plots for Bacteriologic Failure and Adverse Events in All Included Studies. Figure S2. Forest Plots for Subgroup Analyses of Clinical Failure. Figure S3. Funnel Plot of Included Studies for Clinical Failure. Table S1. PRISMA Check List. Table S2. Quality Assessment of Included Studies. Table S3. Characteristics of Excluded Full-Text Articles. (DOC 428 kb) [file 12879_2017_2495_MOESM1_ESM.doc]

Search Strategy

PubMed/MEDLINE, National Library of Medicine (Searched on 12/11/2016)

#1- "Amoxicillin"[Mesh] OR "Ampicillin"[Mesh] OR "Piperacillin"[Mesh] OR "Cefuroxime"[Mesh] OR "cefpodoxime"[Supplementary Concept] OR "cefditoren"[Supplementary Concept] OR "Cefotaxime"[Mesh] OR "Ceftriaxone"[Mesh] OR "cefdinir"[Supplementary Concept] OR "cefepime"[Supplementary Concept] OR "PPI-0903"[Supplementary Concept] OR "Imipenem"[Mesh] OR "meropenem"[Supplementary Concept] OR "ertapenem"[Supplementary Concept] OR "beta-Lactams"[Mesh] OR "Carbapenems"[Mesh] OR "Penicillins"[Mesh] OR "Cephalosporins"[Mesh]

#2- amoxicillin OR ampicillin OR piperacillin OR penicillin* OR cefuroxime OR cefpodoxime OR cefdinir OR cefditoren OR cefotaxime OR ceftriaxone OR cefepime OR ceftaroline OR cephalosporin* OR imipenem OR meropenem OR ertapenem OR Carbapenem* OR beta-lactam*

#3- 1 or 2

#4- "moxifloxacin"[Supplementary Concept] OR "Levofloxacin"[Mesh] OR "gemifloxacin"[Supplementary Concept] OR "Azithromycin"[Mesh] OR "Erythromycin"[Mesh] OR "Clarithromycin"[Mesh] OR "Doxycycline"[Mesh] OR "Macrolides"[Mesh] OR "Fluoroquinolones"[Mesh]

#5- Moxifloxacin OR levofloxacin OR gemifloxacin OR azithromycin OR erythromycin OR clarithromycin OR doxycycline OR macrolide* OR fluoroquinolone*

#6- 4 or 5

#7- 3 and 6

#8- "community acquired pneumonia"

#9- 7 and 8

Embase, Elsevier (Searched on 12/11/2016)

#1- 'levofloxacin'/exp OR 'moxifloxacin'/exp OR 'gemifloxacin'/exp OR 'azithromycin'/exp OR 'clarithromycin'/exp OR 'erythromycin'/exp OR 'doxycycline'/exp OR 'macrolide'/exp OR 'quinoline derived antiinfective agent'/exp

#2- 'community acquired pneumonia'/exp

#3- 'amoxicillin'/exp OR 'ampicillin'/exp OR 'piperacillin'/exp OR 'penicillin derivative'/exp OR 'cefuroxime'/exp OR 'cefpodoxime'/exp OR 'cefdinir'/exp OR 'cefditoren'/exp OR 'cefotaxime'/exp OR 'ceftriaxone'/exp OR 'cefepime'/exp OR 'ceftaroline'/exp OR 'cephalosporin derivative'/exp OR 'imipenem'/exp OR 'meropenem'/exp OR 'ertapenem'/exp OR 'carbapenem'/exp OR 'beta lactam'/exp

#4- 'randomized controlled trial'/exp OR 'single blind procedure'/exp OR 'double blind procedure'/exp OR 'crossover procedure'/exp

#5- #1 AND #2 AND #3 AND #4

Cochrane Library (Searched on 12/11/2016)

#1- Moxifloxacin or levofloxacin or gemifloxacin or azithromycin or erythromycin or clarithromycin or doxycycline or macrolide or fluoroquinolone:ti,ab,kw (Word variations have been searched)

#2- "community acquired pneumonia":ti,ab,kw (Word variations have been searched)

#3- amoxicillin or ampicillin or piperacillin or penicillin* or cefuroxime or cefpodoxime or cefdinir or cefditoren or cefotaxime or ceftriaxone or cefepime or ceftaroline or cephalosporin* or imipenem or meropenem or ertapenem or Carbapenem* or beta-lactam* (Word variations have been searched)

#4- MeSH descriptor: [beta-Lactams] explode all trees

#5- MeSH descriptor: [Macrolides] explode all trees

#6- MeSH descriptor: [Fluoroquinolones] explode all trees

#7- MeSH descriptor: [Doxycycline] explode all trees

#8- #1 or #5 or #6 or #7

#9- #3 or #4

#10- #2 and #8 and #9

**Figure S1. Forest Plots for Bacteriologic Failure and Adverse Events in All Included Studies:**

1. **Bacteriologic Failure**

**
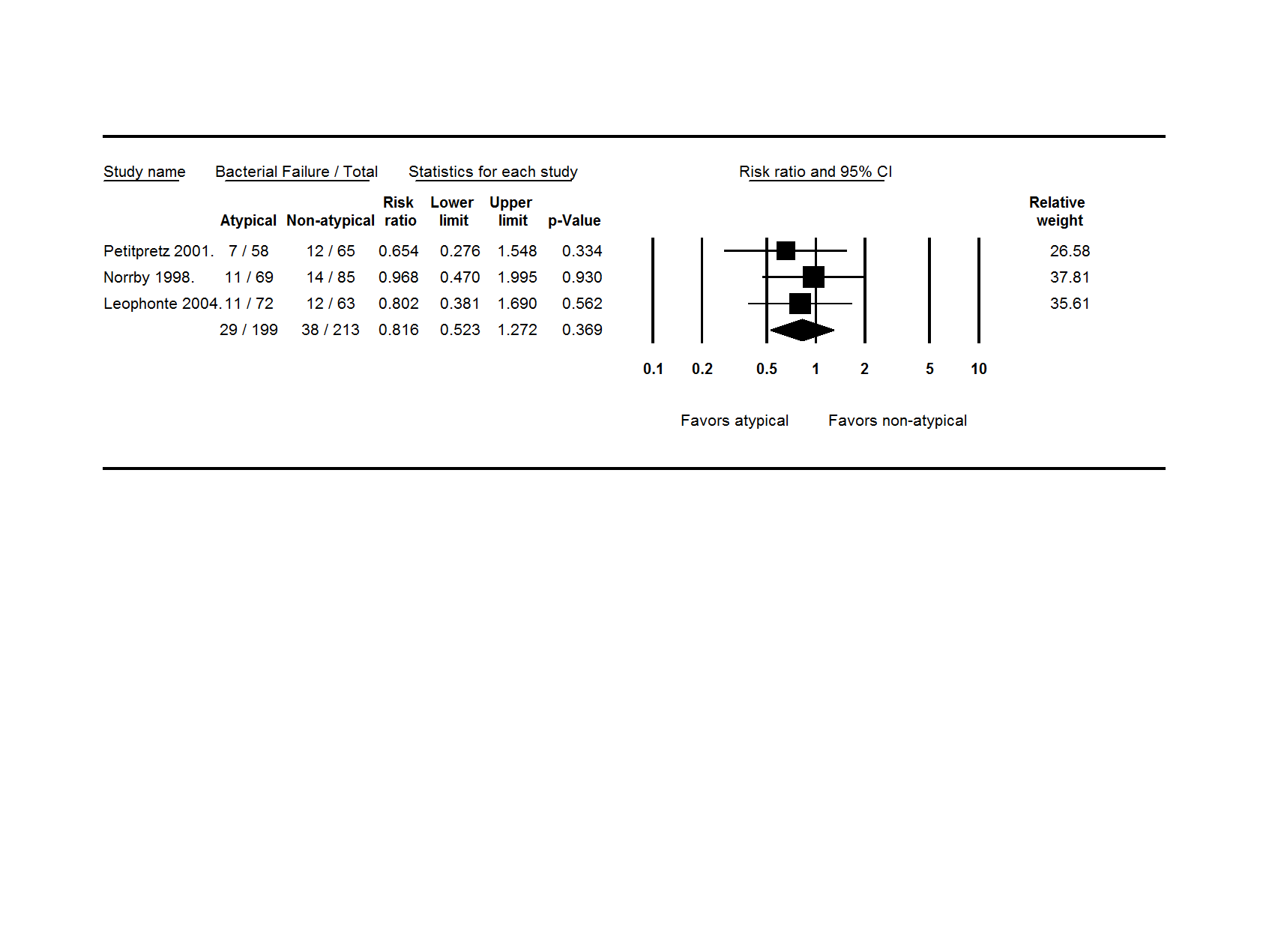
**

1. **Total Adverse Events**

**
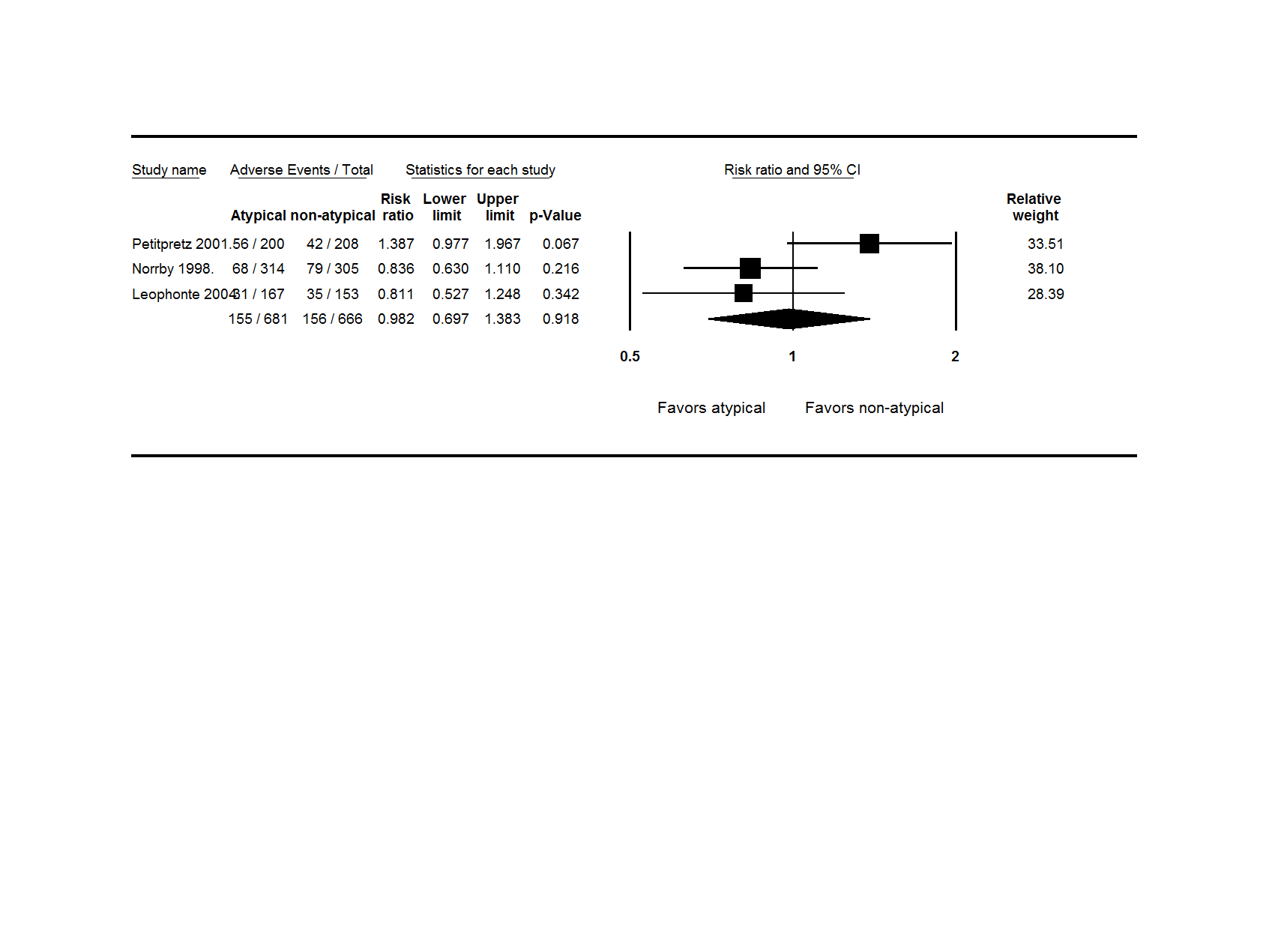
**

1. **Diarrhea**

**
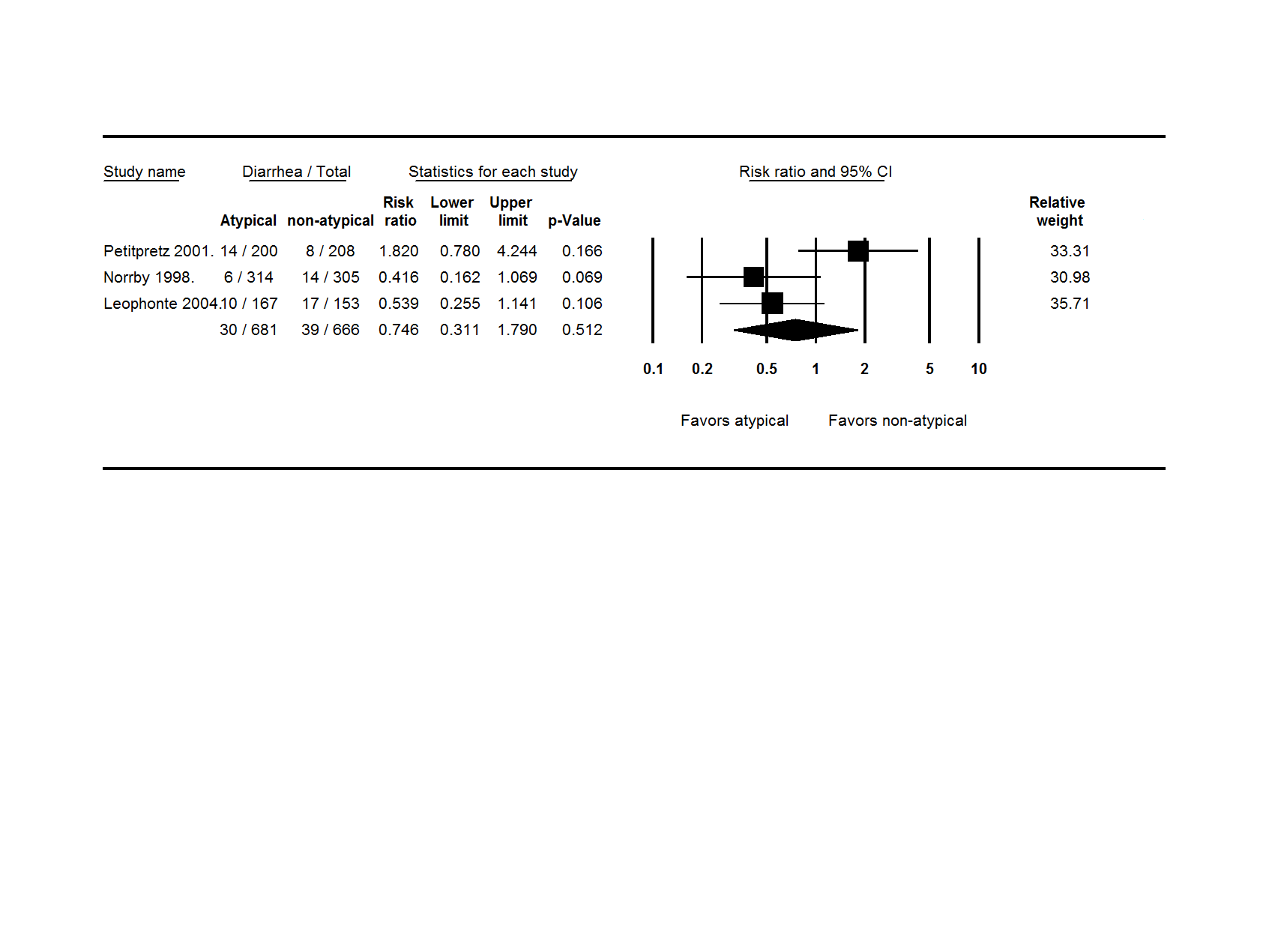
**

1. **Adverse Events Requiring Discontinuation of Antibiotic Therapy**

**
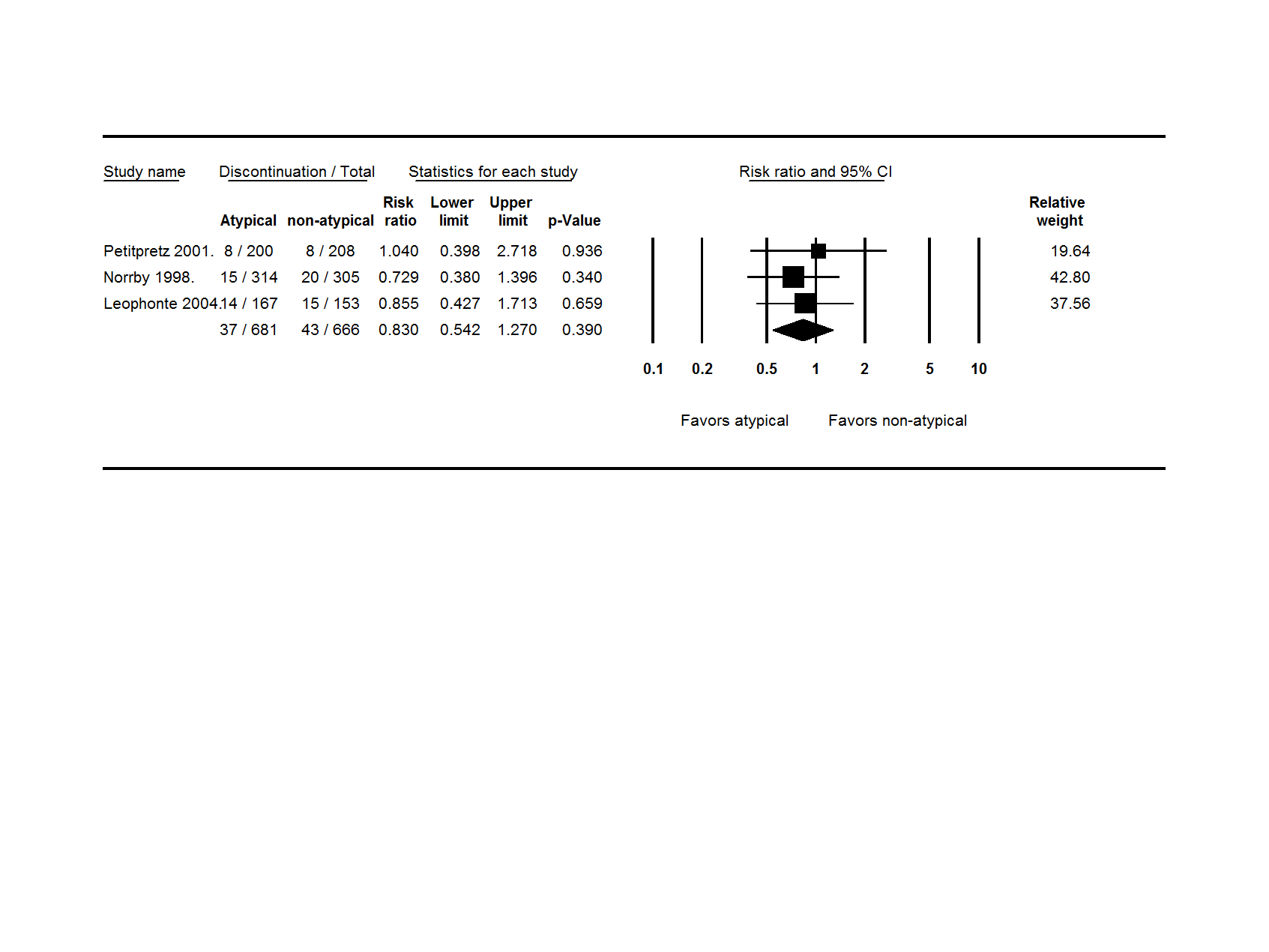
**

**Figure S2. Forest Plots for Subgroup Analyses of Clinical Failure:**

1. **Fluoroquinolones versus Macrolides**

**
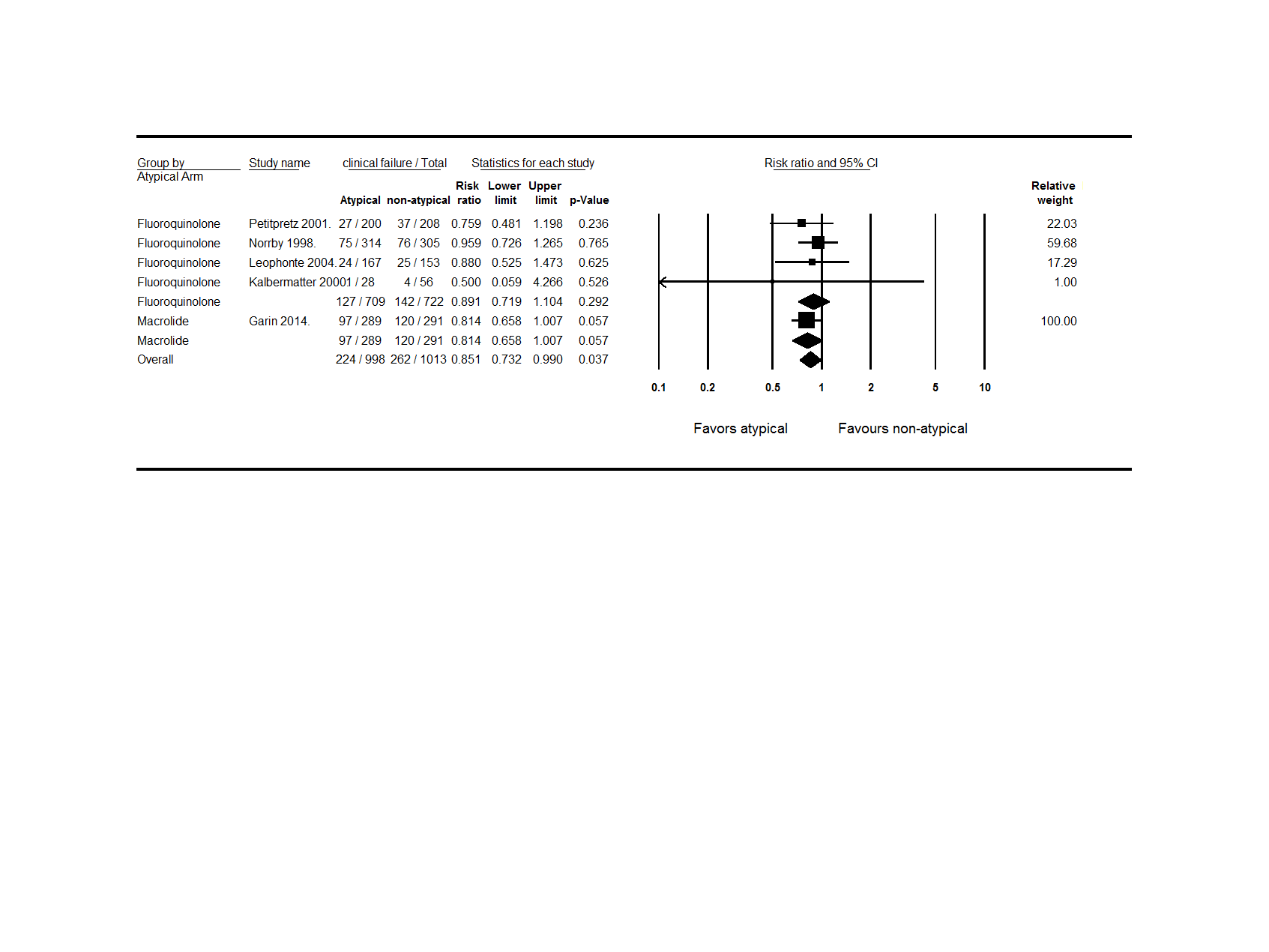
**

1. **Superiority versus Non-Inferiority Studies**

**
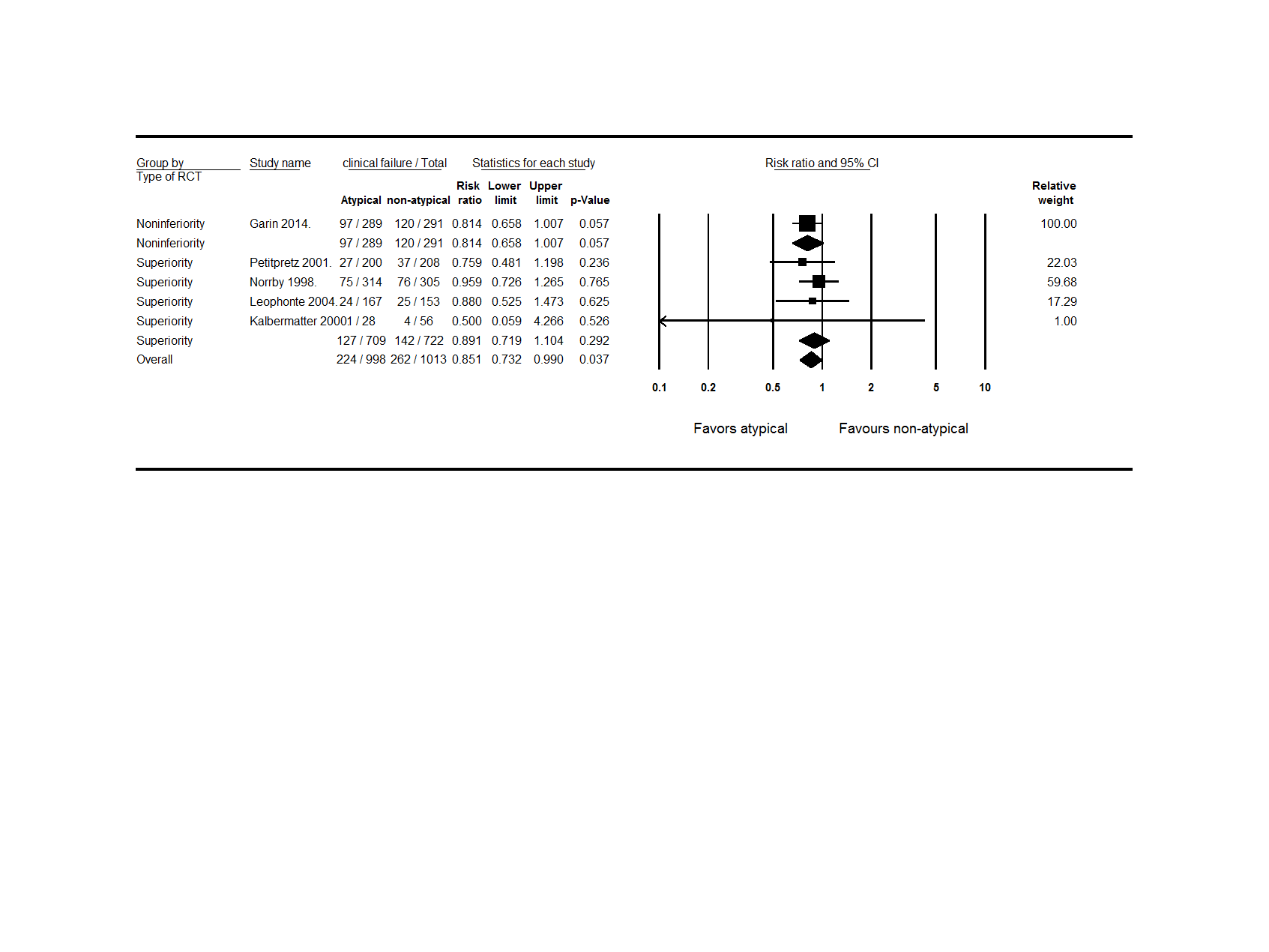
**

1. **Publication Year**

**
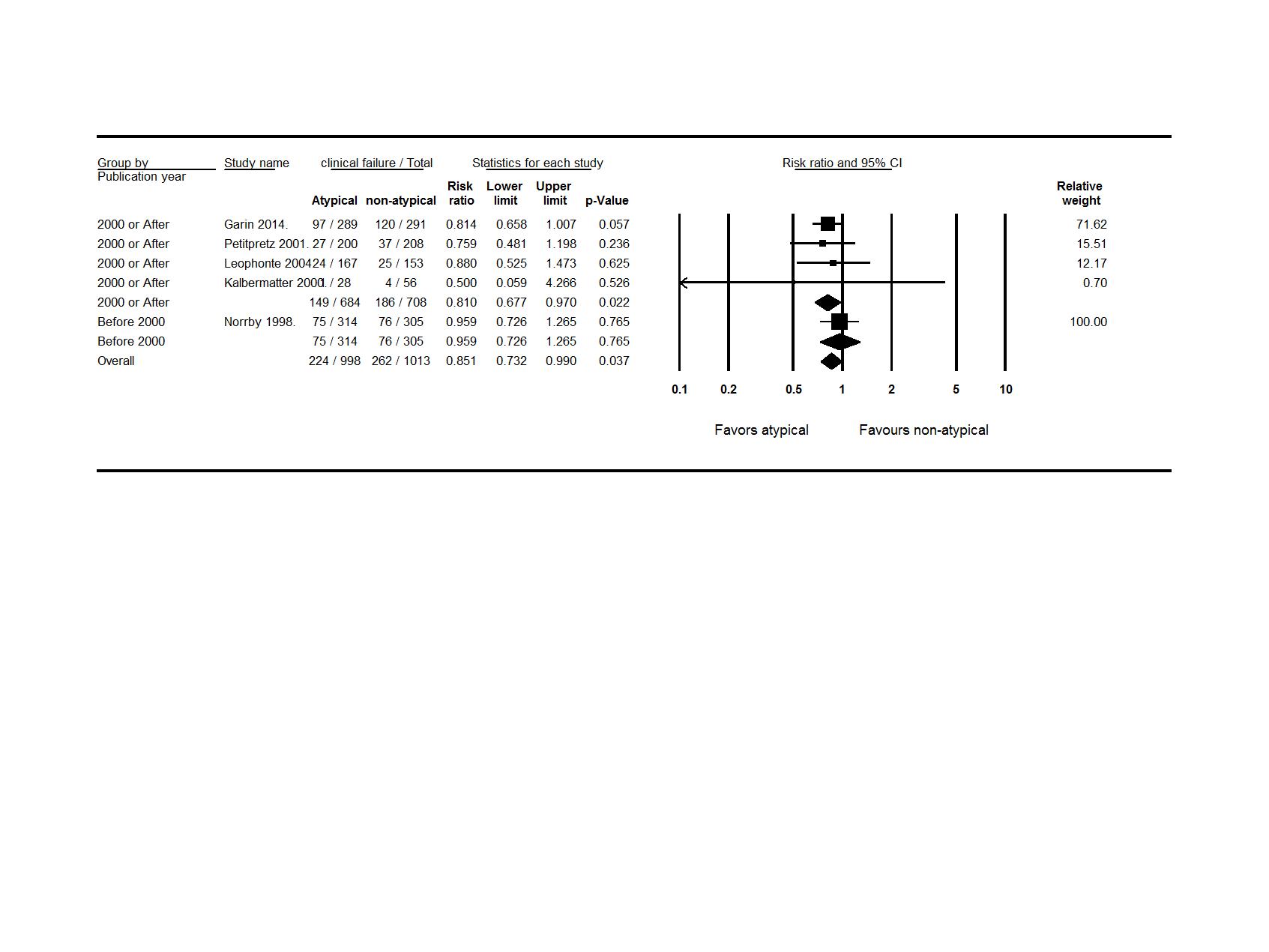
**

1. **Severity of Community-Acquired Pneumonia (CAP)**

**
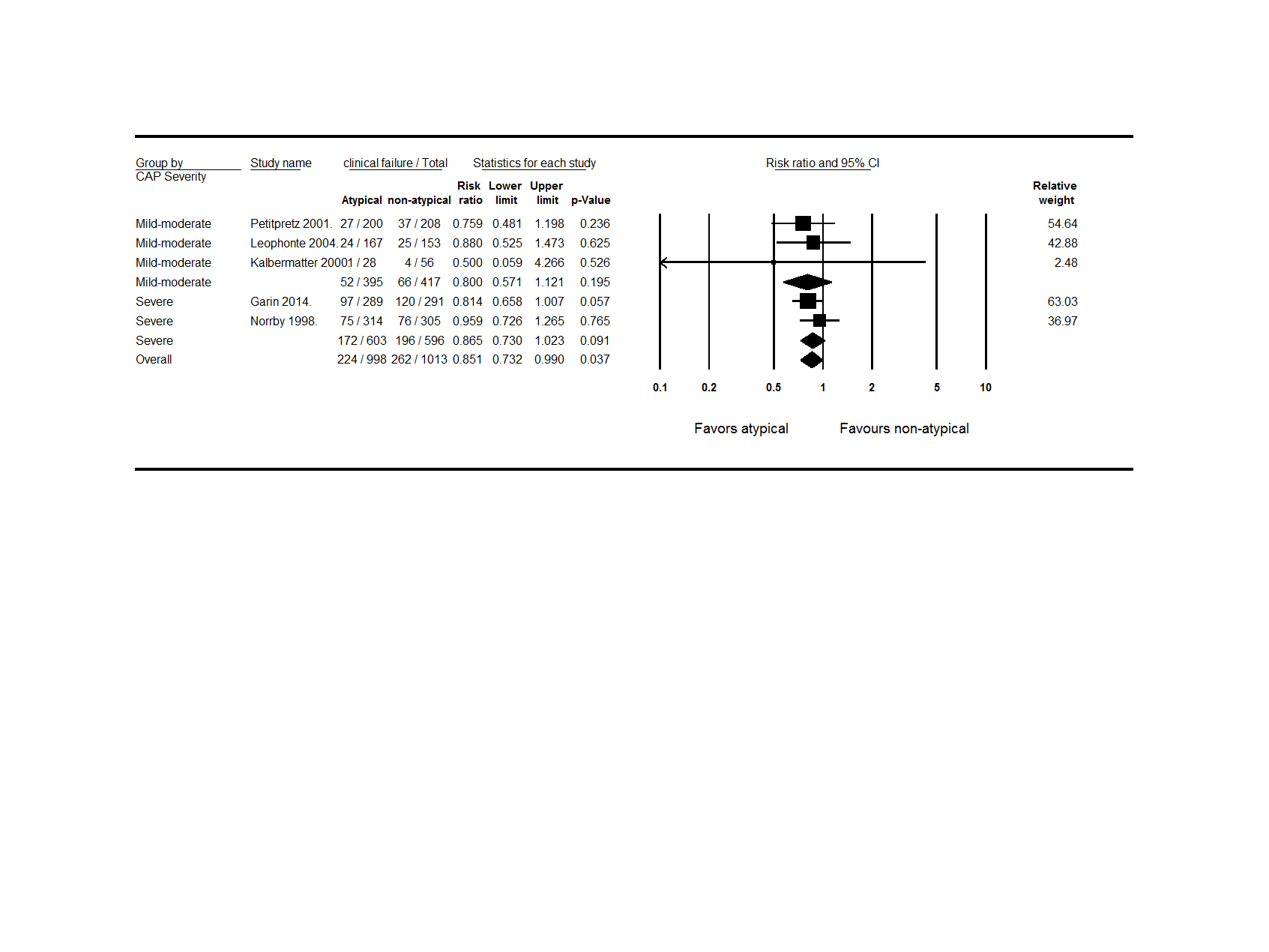
**

**Figure S3. Funnel Plot of Included Studies for Clinical Failure**

**
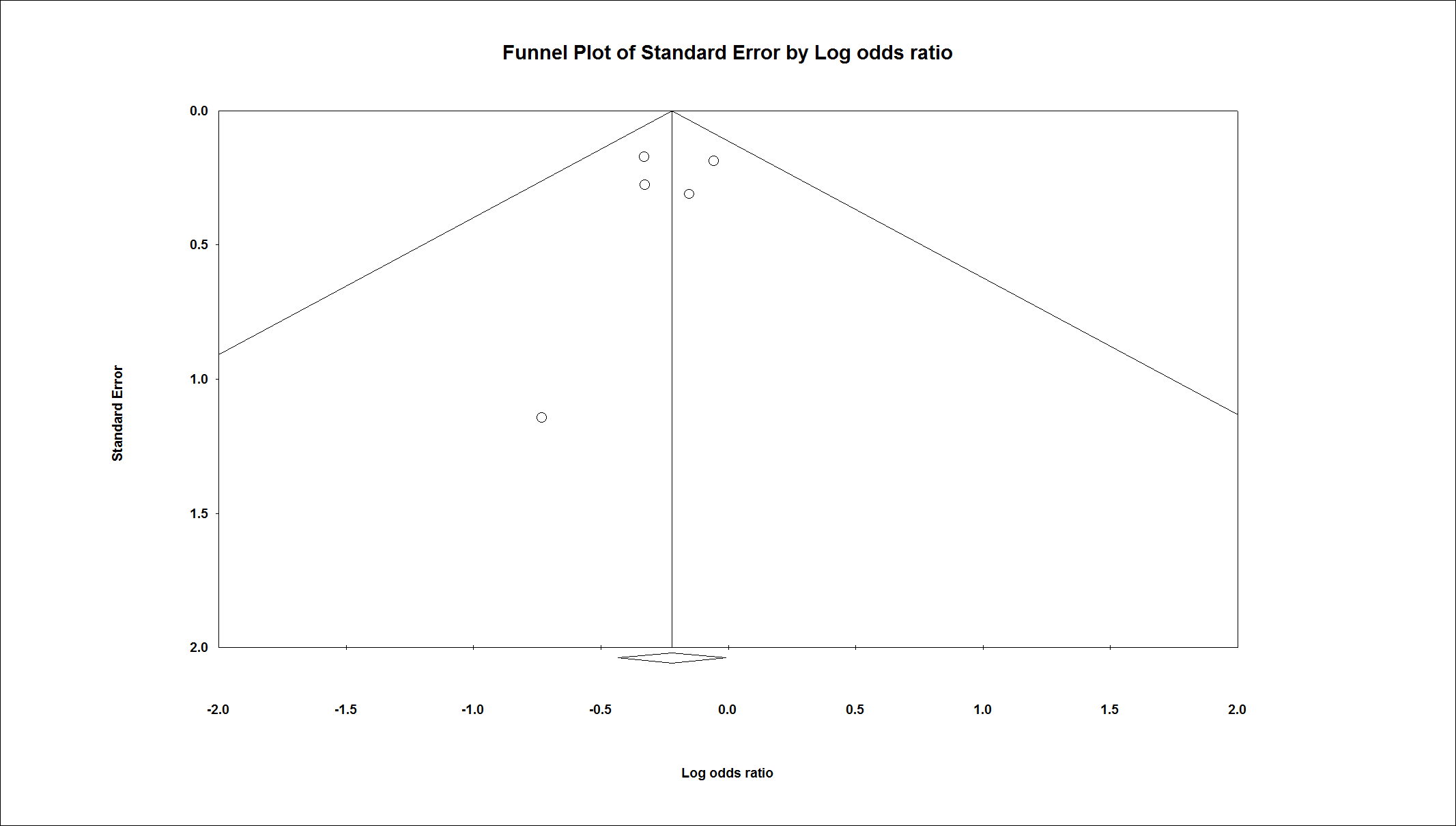
**

**Table S1. PRISMA Check L**ist

| **Section/topic** | **#** | **Checklist item** | **Reported on page #** |
| --- | --- | --- | --- |
| **TITLE** | | |  |
| Title | 1 | Identify the report as a systematic review, meta-analysis, or both. | 1 |
| **ABSTRACT** | | |  |
| Structured summary | 2 | Provide a structured summary including, as applicable: background; objectives; data sources; study eligibility criteria, participants, and interventions; study appraisal and synthesis methods; results; limitations; conclusions and implications of key findings; systematic review registration number. | 3 |
| **INTRODUCTION** | | |  |
| Rationale | 3 | Describe the rationale for the review in the context of what is already known. | 4 |
| Objectives | 4 | Provide an explicit statement of questions being addressed with reference to participants, interventions, comparisons, outcomes, and study design (PICOS). | 4 |
| **METHODS** | | |  |
| Protocol and registration | 5 | Indicate if a review protocol exists, if and where it can be accessed (e.g., Web address), and, if available, provide registration information including registration number. | Not applicable |
| Eligibility criteria | 6 | Specify study characteristics (e.g., PICOS, length of follow-up) and report characteristics (e.g., years considered, language, publication status) used as criteria for eligibility, giving rationale. | 5-6 |
| Information sources | 7 | Describe all information sources (e.g., databases with dates of coverage, contact with study authors to identify additional studies) in the search and date last searched. | 5 |
| Search | 8 | Present full electronic search strategy for at least one database, including any limits used, such that it could be repeated. | Suppl. |
| Study selection | 9 | State the process for selecting studies (i.e., screening, eligibility, included in systematic review, and, if applicable, included in the meta-analysis). | 5-6 |
| Data collection process | 10 | Describe method of data extraction from reports (e.g., piloted forms, independently, in duplicate) and any processes for obtaining and confirming data from investigators. | 5 |
| Data items | 11 | List and define all variables for which data were sought (e.g., PICOS, funding sources) and any assumptions and simplifications made. | 5-6 |
| Risk of bias in individual studies | 12 | Describe methods used for assessing risk of bias of individual studies (including specification of whether this was done at the study or outcome level), and how this information is to be used in any data synthesis. | 6 |
| Summary measures | 13 | State the principal summary measures (e.g., risk ratio, difference in means). | 6 |
| Synthesis of results | 14 | Describe the methods of handling data and combining results of studies, if done, including measures of consistency (e.g., I^2^) for each meta-analysis. | 6 |

| **Section/topic** | **#** | **Checklist item** | **Reported on page #** |
| --- | --- | --- | --- |
| Risk of bias across studies | 15 | Specify any assessment of risk of bias that may affect the cumulative evidence (e.g., publication bias, selective reporting within studies). | 6 |
| Additional analyses | 16 | Describe methods of additional analyses (e.g., sensitivity or subgroup analyses, meta-regression), if done, indicating which were pre-specified. | 10 |
| **RESULTS** | | |  |
| Study selection | 17 | Give numbers of studies screened, assessed for eligibility, and included in the review, with reasons for exclusions at each stage, ideally with a flow diagram. | Flowchart 1 |
| Study characteristics | 18 | For each study, present characteristics for which data were extracted (e.g., study size, PICOS, follow-up period) and provide the citations. | Table 1 |
| Risk of bias within studies | 19 | Present data on risk of bias of each study and, if available, any outcome level assessment (see item 12). | 7(+Suppl.eTable2) |
| Results of individual studies | 20 | For all outcomes considered (benefits or harms), present, for each study: (a) simple summary data for each intervention group (b) effect estimates and confidence intervals, ideally with a forest plot. | Figure 1-2 (Suppl.eFigure1) |
| Synthesis of results | 21 | Present results of each meta-analysis done, including confidence intervals and measures of consistency. | Figure 1-2 (Suppl.eFigure1) |
| Risk of bias across studies | 22 | Present results of any assessment of risk of bias across studies (see Item 15). | 6(+Suppl.eFigure3) |
| Additional analysis | 23 | Give results of additional analyses, if done (e.g., sensitivity or subgroup analyses, meta-regression [see Item 16]). | Suppl.eFigure2 |
| **DISCUSSION** | | |  |
| Summary of evidence | 24 | Summarize the main findings including the strength of evidence for each main outcome; consider their relevance to key groups (e.g., healthcare providers, users, and policy makers). | 8-10 |
| Limitations | 25 | Discuss limitations at study and outcome level (e.g., risk of bias), and at review-level (e.g., incomplete retrieval of identified research, reporting bias). | 10-11 |
| Conclusions | 26 | Provide a general interpretation of the results in the context of other evidence, and implications for future research. | 12 |
| **FUNDING** | | |  |
| Funding | 27 | Describe sources of funding for the systematic review and other support (e.g., supply of data); role of funders for the systematic review. | No funding |

Table S2. Quality Assessment of Included Studies

|  |  | **Selection bias** | | **Performance bias** | **Detection bias** | **Attrition bias** | **Reporting bias** | **Other bias** |
| --- | --- | --- | --- | --- | --- | --- | --- | --- |
| **Study** | **Publication year** | **Random sequence generation** | **Allocation concealment** | **Blinding of participants and personnel** | **Blinding of outcome assessment ^a^** | **Incomplete outcome data** | **Selective reporting** | **Other bias** |
| Norrby et al | 1998 | **-** | **-** | **+** | **?** | **+** | **-** | **?** |
| Kalbermatter et al | 2000 | **?** | **?** | **+** | **+** | **-** | **-** | **?** |
| Petitpretz et al | 2001 | **-** | **-** | **-** | **-** | **+** | **-** | **?** |
| Leophonte et al | 2004 | **?** | **?** | **-** | **-** | + | **-** | **?** |
| Garin et al | 2014 | **-** | **-** | **+** | **-** | **-** | **-** | **?** |

^a^For both clinical failure and mortality. +, low risk of bias; “?” Unclear risk of bias; “-” high risk of bias

Table S3. Characteristics of Excluded Full-Text Articles

| Excluded Study | Reason of Exclusion |
| --- | --- |
| Badaro 2002 | No pure empiric atypical coverage arm |
| Carbon 1999 | Unknown proportion of hospitalized patients |
| File 1997 | Insufficient or proportion of hospitalized patients |
| Finch 2002 | Allowed atypical coverage in the empiric non-atypical coverage arm |
| Garin 2014 | Duplicate publication |
| Geijo 2002 | Allowed atypical coverage in the empiric non-atypical coverage arm |
| Hatipoglu 2010 | Abstract |
| Katz 2004 | Allowed atypical coverage in the empiric non-atypical coverage arm |
| Kohno 2011 | Language of publication (Japanese) |
| Lode 2002 | Allowed atypical coverage in the empiric non-atypical coverage arm |
| Postma 2015 | Allowed atypical coverage in the empiric non-atypical coverage arm |
